# Finite Element Evaluation of Stress Distribution in Mature and Immature Teeth

**Published:** 2007-07-05

**Authors:** Ali Talati, Reza Disfani, Ashkan Afshar, Akbar Fallah Rastegar

**Affiliations:** 1 *Department of Endodontics, Dental School and Dental Research Center, Mashad University of Medical Sciences, Mashad, Iran*; 2 *Department of Endodontics, Dental School, Rafsanjan University of Medical Sciences, Rafsanjan, Iran*; 3 *Department of Endodontics, Dental School, Mashad University of Medical Sciences, Mashad, Iran*

**Keywords:** Finite Element, Immature Apex, Traumatic Injury

## Abstract

**INTRODUCTION:** Traumatic injuries may affect the vitality and development of tooth. So divergence or parallelism of canal walls induces an open apex which is very susceptible to fracture. The aim of this study was to determine the stress distribution pattern in immature teeth and compare it with mature teeth.

**MATERIALS AND METHODS:** In order to analyze stress distribution using finite element method, models were first designed according to actual samples by "ANSYS" software. Two models of the maxillary central incisors were designed, one mature and one with open apex. Mature tooth was designed as having undergone root canal therapy with gutta-percha and the immature one with both MTA plug and gutta-percha. Samples were loaded in seven stages and then, the stress distribution in each model was measured, using the "ANSYS" software.

**RESULTS:** During gutta-percha condensation, the immature tooth transfers the stress directly to the external root surface where force is directly applied, whereas during masticatory occlusal forces, the stress is transferred to middle third of buccal and lingual surfaces and to buccal cervix in lunar shape. During traumatic forces, stress concentration was on the cervical region of buccal surface and middle third of buccal and lingual surfaces. MTA plug prevents stress distribution toward apical region and forces concentrate in dentin at the point where stress terminates.

**CONCLUSION:** Within the limitations of this simulated mechanical analysis, it was confirmed that the pattern of stress distribution in mature and immature teeth is different. Cervical area of the buccal surface in immature teeth is one of the stress concentration areas, which contribute to the high rate of fracture in this area.

## INTRODUCTION

The most accident-prone time period in dental injuries is in age range of 8 to 12 years in which maxillary central incisors are the most frequently involved teeth. Traumatic incidences in this period may affect the vitality and root development of tooth (1-2), so divergence or parallelism of the canal walls induces an open apex, which is very susceptible to fracture (2-4). In fact, 30% of these teeth would be fractured during or after endodontic treatment (5). For treatment of these teeth, two protocols have been proposed which include apexifica-tion and apical plug, both of which have high rate of success (3-4).

Several studies have analyzed the stress distribution in endodontically treated teeth. These studies have been performed using experimental methods such as strain-gauge measurements (6-7), photo elastic techniques (8), and finite element method (FEM) (9).

FEM is an engineering method for the numerical analysis of structural based material properties. Material properties, such as Poissant Ratio and the modulus of elasticity can be utilized by computer-generated analyses to describe the mechanical behavior of a structure. Therefore, any simple or complex structure can be modeled with the computer through the use of nodes and elements, which describe structures as a collection of many smaller subsections. A node is a coordinate in space where degrees of freedom (displacements) and actions (forces) of a structure under load are considered to exist. An element is a mathema-tical matrix of the collective interaction among degrees of freedom between a set of nodes. Once a structure is numerically created and material properties are assigned, it can be analyzed for stress distributions during force application using FEM (9-10).

Advantages of FEM that can be adapted to its use in dentistry include accurate modeling of complex geometry, systematic model modifica-tion, and analysis of the stress-strain patterns. Having many limiting assumptions and validation of results, FEM is not always experi-mentally easy. However, direct measurement of stresses and strains in root canal walls are impractical. The distribution of stresses is likely to be complex and the internal canal wall is inaccessible for measuring devices such as strain gauges (11).

The aim of this study was to determine the stress distribution pattern in immature teeth using FEM, and to compare it with mature teeth for detection of this pattern effect on the fracture susceptibility.

## MATERIALS AND METHODS

Three-dimensional FEM was used to perform the stress analysis of the tooth. This method is particularly suitable to biological structure analysis as it allows great flexibility in dealing with geometric complex domains composed by multiple materials.

The upper central incisor was selected because of its single root with relatively simple anatomy and highly susceptibility to fracture. Two models of a maxillary central incisor were designed, one with open apex and the other with mature apex. Tooth materials were assumed to be isotropic, homogeneous, and elastic. Dimensions and morphology of the tooth in addition to Poissont ratio, and the modulus elasticity of cancellous bone, cortical bone, enamel, dentin, PDL, gutta-percha, and composite resin were constructed based on dimensions obtained from the literatures (12-13). The geometry of the mature tooth was obtained from Woelfel (12). The diameter of the apical portion (termination) was a size #40 file, with a coronal orifice diameter of 1.60 mm (10). The canal was fabricated in a taper form to have a thorough simulation of an actual prepared canal. Modeling of the open apex tooth was impossible because the actual dimensions of such teeth have not been mentioned before. Resultantly, two parallel radiographs in two dimensions (buccolingual and mesiodistal) were taken from an extracted open apex tooth which had parallel dentinal walls in the apical portion of the canal. After scanning of the radiographs, modeling and dimension determination was applied by AutoCad software program (Auto Desk Inc., San Raphael, California).

Because of the specific goal of the study which was the evaluation of stress distribution in endodontically treated teeth, the canal space in mature tooth was obturated with gutta-percha from 1 mm above the anatomic apex up to 1 mm under CEJ. In immature tooth, 4 mm MTA plug was placed in the apical portion and then remaining of the canal up to 1 mm under CEJ was obturated with gutta-percha. Finally, the access cavity was filled with composite resin in both models.

The analysis was accomplished using specific software, ANSYS Primer for Stress Analysis, revision 5.4 (Swanson Analysis System, Inc., Houston, PA, USA).

A simultaneous combination of the numeric amount of stresses (in X,Y and Z axes) is called Von Mises stresses (9) which show the location of the most highly stressed areas, with no possibility of determining the nature (tensile or compressive) of the stress (14). Von Mises criterion was used in this study for the comparison between open apex and mature teeth.

**Figure 1:**
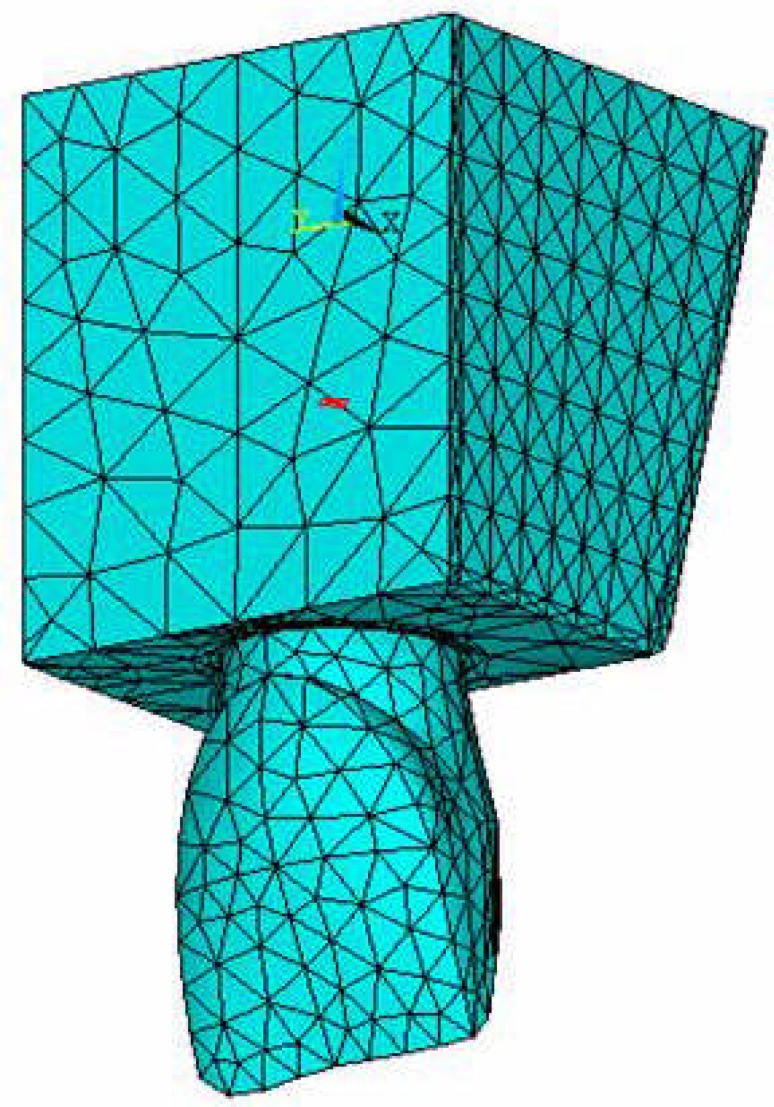
Element determination of the central model

In all FEMs, the domain is divided into a finite number of elements. These elements are connected at points called nodes. In solids models, displacements in each element are directly related to the nodal displacements. The nodal displacements are then related to the strains and the stresses in the elements. FEM tries to choose the nodal displacements so that the stresses are in equilibrium with the applied loads. It converts the conditions of equilibrium into a set of linear algebraic equations for the nodal displacements. By breaking the structure into a numerous smaller elements, the stresses become closer to achieving equilibrium with the applied loads. The number and size of elements appoints the accuracy of analysis, so in PDL and root dentinal areas which require more accurate analysis, smaller elements were chosen (Figure 1).

The evaluation of the effect of applied forces on the stress pattern was done in two situations during and after the endodontic treatment:

During endodontic treatment, each tooth was loaded in three stages which were included the load of gutta-percha condensation in the apical, middle, and coronal thirds of the root, respectively.

It is known that clinical conditions resemble loading ranging from 10 to 30N. To be more critical in this study, the known maximum loading has been increased by 50%, and a 45N vertical load was applied in the analysis (15). In open apex tooth, because of the application of 4mm MTA plug, the remainder space of the canal was divided into 3 equal sections and each of these gutta-percha sections were loaded separately.

**Table 1:** The amount of maximum stress in Gutta-Percha vertical condensation in different canal thirds

**Apex**	**surface of root dentin**	**Apical**	**Middle**	**Coronal**
Complete	External	6.5	1.8	3.5
	Internal	55	85	45
Open	External	6.9	3.2	2.5
	Internal	12	10	8

After completion of endodontic treatment and loading in oral cavity, two possible forces will affect the tooth which includes: A) Occlusal load during function and B) load of possibly later traumatic force to the crown.

A) A static load of 100N was applied at 45 degree inclination with respect to the near of incisor’s edge in the palatal surface. This degree tilted with respect to the tooth’s longitudinal axis in order to simulate the centric occlusal contact with the opposite tooth (16).

B) The crown received perpendicular occlusal load to the buccal surface at intensity of 100, 200, and 300N, respectively.

Collectively, each sample was loaded in seven stages: condensation load of gutta-percha in the apical, middle, and coronal thirds of the root, masticatory occlusal load to the lingual surface, and traumatic load to the buccal surface at intensity of 100, 200, and 300N, respectively.

## RESULTS

**A)** The comparison of stress distribution in dentin of mature and immature teeth with three loading patterns for simulation: 

**1**) The stress distribution pattern following vertical condensation of gutta-percha 

The numerical results obtained from vertical condensation of gutta-percha in various parts of the root have been summarized in Table 1.

***1-a )*** Apical third**: **If apex is mature, the stresses due to the condensation load in the apical third surrounds the condensation area (between apical and middle third) in the internal area of dentin in a ring shape, whereas the stress loaded to the external area is in the mesial and distal surface of the apical third. In immature apex which had received MTA plug, the stresses surround the condensation area in the internal area in a ring shape but the stress loaded to the external area is actually parallel to the inner condensation area.

**Figure 2:**
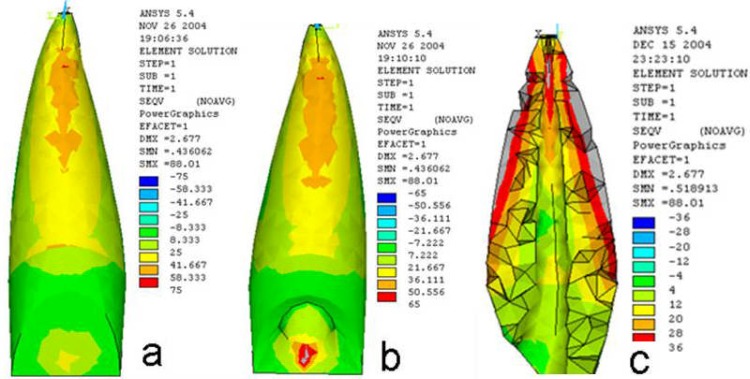
Stress distribution pattern in dentin of the mature tooth a) buccal surface b) lingual surface c) internal and external surfaces following the masticatory forces.

***1-b )*** Middle third: If apex is mature, the stresses surround the condensation area (between middle and coronal third) in the internal area of dentin in a ring shape, whereas the stress loaded to the external area is in the mesial and distal surfaces of the middle third. In immature apex which has received MTA plug, the stress pattern in the internal area is similar to mature tooth, but in the external area is actually parallel to the condensation area.

***1-c )*** Coronal third**: **If apex is mature, the stresses surround the gutta-percha in the internal area of the coronal dentin in a ring shape, but the stress loaded to the external area is in the mesial and distal surfaces of the coronal third. In immature apex which has received MTA plug, the stress pattern in the internal area is similar to the mature tooth, but in the external area is actually parallel to the condensation area (coronal third).

**2)** The stress distribution following masticatory occlusal forces have been summarized in Table 2.

**Table 2:** The amount of maximum stress following mastication occlusal farces in open apex and complete apex teeth.

**surface of ** **root dentin**	**Complete apex**		**Open apex**
External	Buccal	78	60
	Lingual	65	50
Internal	50		60

In mature tooth, the stress was mostly concentrated in external area of the apical third, obviously in the buccal and lingual surfaces, whereas in the internal area, it was mostly concentrated in the apical third, obviously in the mesial and distal surfaces (Figure 2).

**Figure 3:**
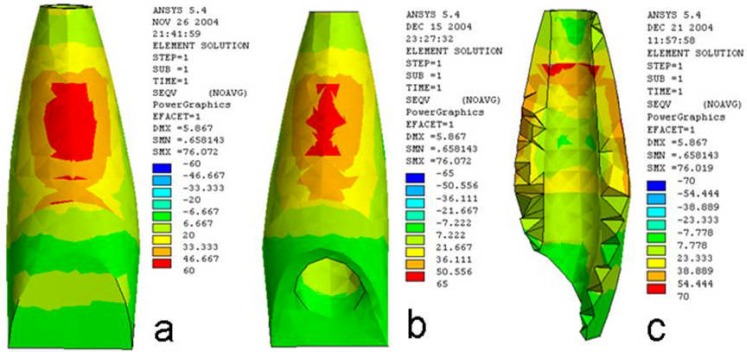
Stress distribution pattern in dentin of the immature tooth a) in the buccal surface b) in the lingual surface c) in the internal & external surfaces following the mastication occlusal forces.

In immature tooth which was received MTA plug, the stress was mostly concentrated in the external area of the middle third of the buccal and lingual surfaces and also to the buccal cervix in a lunar shape, whereas in the internal area, it was mostly concentrated in termination point of plug (Figure 3).

**3)** The stress distribution following direct traumatic forces to the buccal surface has been summarized in table 3.

**Table 3:** The amount of maximum stress concentration points following application of 100,200 and 300N direct trauma.

**Apex**	**surface of** **root dentin**	**100N**		**200N**	**300N**
Close	External	Buccal	60	118	175
		Lingual	55	110	162
	Internal		37	80	120
Open	External	Buccal	37	74	110
		Lingual	40	80	123
	Internal		140	96	45

100N force in mature tooth, distributed the stress on the entire buccal and lingual external area of the root in an elongated ellipse shape with maximum concentration in the apical third. However, immature tooth revealed the concentration of stress on the cervical region of the buccal surface and the middle third of the buccal and lingual surfaces. The pattern in the internal area was a line parallel to the longitudinal axis of root in the apical third of the mesial and distal surfaces.

In open apex tooth, stress distribution had a two-stage pattern in external area in buccal surface. In cervical area, it was in a lunar shape, whereas in the middle third, it was in elliptical-circular shape. The pattern in the lingual surface was similar to this; the only difference was conjugation of the cervical luna by middle ellipse. In the inner dentin, stress concentration had different pattern in which, the concen-tration area of stress was in the buccal and lingual borders of the ending point of plug.

The pattern of stress distribution due to 200 and 300N traumatic forces was similar to 100N force, but the quantity of stress was greater.

**B)** The pattern of stress distribution in the MTA plug in all of seven situations was equal but the numerical quantity of the maximum stress was different:

Vertical condensation of gutta-percha in apical, middle, and coronal third were done with 18, 8.8, 10 MPa, Masticatory occlusal force with 185 MPa, and direct traumatic force to the buccal surface with 120, 240, 360 MPa.

Maximum load is in the surface of MTA especially in the region of contact with dentinal walls, but in deeper areas of plug, load is rapidly decreased.

## DISCUSSION

The comparison of the teeth fragility is often made by Instron machine which applies a controlled force to the tooth until a fracture happens, but it does not provide any further information about the stress distribution pattern throughout the object and it only indicates the amount of applied force (13).

Furthermore, immature teeth are not easily available because such teeth are not usually extracted in clinic. The simulation of the immature tooth by cutting off the apex and trimming of the canal walls of a mature tooth will not provide the natural situation. According to this reason, the accomplishment of this in vitro investigation is very difficult and the stress distribution pattern is not determined. So, we studied the stress distribution with FEM, using a computerized design of immature tooth.

FEM has some limitations. In spite of the significant advances that have been made in developing FEM packages, the results obtained must be carefully examined before they can be used. The most significant limitation is that the accuracy of the obtained solution is usually a function of the mesh resolution. Any regions of highly concentrated stress, such as around loading points and supports, must be carefully analyzed with the use of a sufficiently refined mesh. In addition, there are some problems which are inherently singular (the stresses are theoretically infinite). An additional concern is that because current packages can solve so many sophisticated problems, there is a strong temptation to solve problems without doing hard work of thinking through them and understanding the underlying mechanics (17).

Although numerical results can be easily obtained in two-dimensional modeling, it has some significant shortcomings: the human tooth is highly irregular, such that, it can not be presented in a two-dimensional space and the actual loading can not be simulated without taking the third dimension into consideration. The distribution of various materials of the tooth structure does not show any symmetry. Therefore, a three-dimensional modeling with the actual dimensions should be preferred for a reliable analysis (10). In this study, a three-dimensional modeling was used.

Takahashi *et al.*, using FEM, showed that the horizontal vector of load was more influential to teeth than the vertical vector (18).

Darendeliler *et al*. designed a study in order to determine the stress distribution with FE. They showed that the compressive stress in dentin was increased from incisal edge to the cervical region which had the most stress. Ending of enamel at the cervical area in addition to the bending effect of the crown causes the accumulation of forces in the cervical area (10).

Ricks-Williamson *et al*., using FEM, invest-tigated the changes in stress characteristics of tooth after simulated canal preparation. The highest stress magnitudes were located between the middle and coronal thirds of the root; an area clinically observed to be prone to fracture during treatment (9).

Yaman *et al.* in a FE study revealed that the likelihood of root fracture due to the vertical condensation technique is a remote possibility (15). Also, Telli and Gulkan, using FEM, concluded that the maximum stress in the vertical condensation technique was in dentin near the gutta-percha which is under compaction (19). In the present study, vertical condensation force was less than limit can induce dentinal fracture (in both mature and immature teeth), which is in agreement with Yaman *et al*. (15) and Telli *et al*. (19).

Analysis of the stress distribution following masticatory occlusal force in immature tooth reveals that the more intense stresses appeared in the cervical region. In addition to thinning of dentinal walls in this area, an important cause is the pattern of stress distribution. In immature tooth, load concentration is in the ending of plug and is perpendicular to the long axis. The pattern of stress distribution in inner and outer areas of dentin is probably because of difference between mature and immature tooth in anatomy of the root and PDL and also the presence of plug.

The presence of MTA plug was effective in the stress distribution pattern because it did not permit transferring of stress in the inner and outer canal walls. In fact, the initiation point of MTA was the ending point of stress. MTA plug induces the concentration of stress in the wall adjacent to it. This is a weakling point in immature tooth which has been treated by MTA plug. No stress distribution from the border of MTA is because of its high elasticity modulus compared to other dental materials. Elasticity modulus of MTA is 2.06x10^5^ which is twice more than enamel. This high elasticity modulus cause its stiffness; so when in contact with dentin by 1.86x10^4 ^elasticity modulus, does not let the stress for suitable distribution. Prevention from apical transferring of stress causes a modification in distribution pattern. It changes from parallel to the long axis of the root to concentration in the middle and coronal third.

Results of this study, present two stress concentration areas which are weakling points of immature tooth with MTA plug. These include bucco-cervical area and the ending point of MTA plug. These areas are very susceptible to fracture. Because of the firm structure of MTA, in addition to rapid decrease of loads in depth of plug, it seems that the possibility of cracking in MTA and losing of sealing ability in the common range of forces is not very high.

Resultantly, saving the pulpal vitality (apexogenesis) is very critical in open apex teeth. In the case of complete devitalized pulp, apexification is preferred to MTA plug.

## CONCLUSION

Within the limitations of this simulated mechanical analysis, it was confirmed that the pattern of stress distribution in mature and immature teeth is different. Application of MTA plug in immature teeth can increase this difference. Cervical area of the buccal surface in immature teeth is one of the stress concentration areas which contribute to the high rate of fracture of this area. In every situation, the mature teeth transfer the load to the apical third of the root and distribute it in a wide surface; but in immature teeth, this area is smaller and MTA plug has a major effect in limitation of the stress distribution area.
